# Correction: Identification of RegIV as a Novel GLI1 Target Gene in Human Pancreatic Cancer

**DOI:** 10.1371/annotation/e8ca8d86-aa97-4951-8359-f8943c1dd935

**Published:** 2011-04-27

**Authors:** Feng Wang, Ling Xu, Chuanyong Guo, Aiwu Ke, Guoyong Hu, Xuanfu Xu, Wenhui Mo, Lijuan Yang, Yinshi Huang, Shanshan He, Xingpeng Wang

The grant number for "National Nature Science Foundation of Education of China" is incorrect. The correct grant number is: "(No.81072005)."

